# Antimutagenic and Anticarcinogenic Evidence of the Genus *Ficus* L.

**DOI:** 10.3390/plants15040654

**Published:** 2026-02-20

**Authors:** Eduardo Madrigal-Santillán, Jacqueline Portillo-Reyes, Luis Fernando García-Melo, José A. Morales-González, Marvin Antonio Soriano Ursúa, Manuel Sánchez-Gutiérrez, Jeannett A. Izquierdo-Vega, Aleli Julieta Izquierdo-Vega, Isela Álvarez-González, Ángel Morales-González, Nikola Batina, Eduardo Madrigal-Bujaidar

**Affiliations:** 1 Escuela Superior de Medicina, Instituto Politécnico Nacional, Ciudad de México 11340, Mexico; jmorales101@yahoo.com.mx (J.A.M.-G.); soum13mx@gmail.com (M.A.S.U.); 2 Escuela Nacional de Medicina y Homeopatía, Instituto Politécnico Nacional, Ciudad de México 07320, Mexico; jacke_star230990@hotmail.com; 3 Laboratorio de Nanotecnología e Ingeniería Molecular, Universidad Autónoma Metropolitana-Iztapalapa, Ciudad de México 09340, Mexico; electronicfer@hotmail.com (L.F.G.-M.); bani@xanum.uam.mx (N.B.); 4 Instituto de Ciencias de la Salud, Universidad Autónoma del Estado de Hidalgo, Pachuca de Soto 42080, Mexico; spmtz68@yahoo.com.mx (M.S.-G.); ivega@uaeh.edu.mx (J.A.I.-V.); aleli_izquierdo11168@uaeh.edu.mx (A.J.I.-V.); 5 Escuela Nacional de Ciencias Biológicas, Instituto Politécnico Nacional, Ciudad de México 07738, Mexico; isela.alvarez@gmail.com; 6 Escuela Superior de Cómputo, Instituto Politécnico Nacional, Ciudad de México 07738, Mexico; anmorales@ipn.mx

**Keywords:** antioxidant, bioactive compounds, *Ficus* plants, genoprotective potential

## Abstract

Among the most important species of *Ficus* L. genus are *F. deltoidea*, *F. exasperata*, *F. sycomorus*, *F. religiosa*, *F. microcarpa*, *F. hirta* Vahl., *F. benghalensis*, *F. racemosa*, *F. elastica*, and *F. carica*. The genus has more than 30 traditional ethnomedicinal uses, attributed to the combination of different bioactive compounds, including flavonoids, (flavanols, flavones, flavonols, isoflavones, chalcones, anthocyanins), phenolic acids (hydroxycinnamic acids, hydroxybenzoic acids), terpenes (triterpenes, tetraterpenes, diterpenes, sesquiterpenes, monoterpenes), phytosterols, coumarins, hydroxybenzoates, phenylpropanoids, chlorins, pheophytins, megastigmans, chitinases, organic acids, fatty acids, amino acids, alkaloids, and glycosides. With this in mind, the objective of this manuscript was to conduct a scientific search in the main electronic databases (PubMed, SciELO, Latindex, Redalyc, BiologyBrowser, ScienceResearch, ScienceDirect, World Wide Science, Web of Science, Academic Journals, Etnobotany, Scopus, and Google Scholar) to gather information on published research regarding the genoprotective potential of the *Ficus* L. genus. Unlike most scientific articles, which primarily describe the individual characteristics and properties of each species, this document compiles the largest number of studies (in vitro and in vivo) on *Ficus* plants described by different authors. Thus, we aim to promote more detailed scientific research and expand studies on the protective capacity of these angiosperm plants to the genetic material.

## 1. Introduction

Despite the great efforts made in primary prevention, many chronic degenerative diseases (currently called noncommunicable diseases, “NCDs”) such as cardiovascular diseases, neuropsychiatric disorders, metabolic diseases (diabetes, obesity), respiratory diseases and cancer, continue to generate high rates of morbidity and mortality worldwide [1]. Numerous epidemiological studies have suggested that a high and adequate consumption of fruits and vegetables can counteract the development of NCDs due to the different bioactive molecules (also called phytochemicals) they contain. Natural bioactive compounds such as flavonoids, phenolic acids, phytosterols, terpenes, and coumarins are recognized for their chemopreventive functions in the incidence of NCDs, and have therefore been proposed as possible therapeutic agents opposing these diseases [2,3,4,5,6].

In the case of cancer, various lines of evidence agree that mutagenesis (induction of permanent changes, “mutations” in DNA) is a fundamental mechanism of carcinogenesis, and although there is a close relationship between carcinogenesis and mutagenesis, they are not identical processes. In this regard, it is worth remembering that many carcinogenic agents induce mutations in genes (oncogenes and anti-oncogenes) that control cell growth and division. However, some carcinogens (non-mutagenic agents) induce the disease through other mechanisms, such as increasing cell division without directly damaging DNA [2,3,4,5,6].

**Figure 1:**
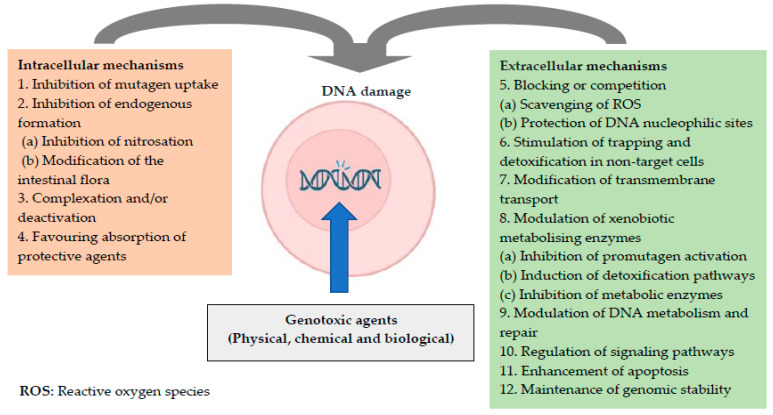
Mechanisms of action of antigenotoxic agents [5,6].

Similarly, it has been suggested that the daily use and/or consumption of antimutagens and anticancer agents (collectively known as antigenotoxic agents) may be an effective method for preventing genetic diseases in humans, including cancer. In general, the mechanisms of action of these agents are complex and are classified as intracellular and/or extracellular; that is, according to the site of action or the specific type of action (Figure 1) [5,6].

Over the past few decades, numerous studies have been conducted to identify new bioactive antigenotoxic molecules that can protect DNA. This field of research focuses on identifying the most effective phytoantimutagens present in the vast biodiversity of fruits, vegetables, as well as edible and/or medicinal plants worldwide [4,5,6].

## 2. *Ficus* L. Genus

### Overview

**Figure 2:**
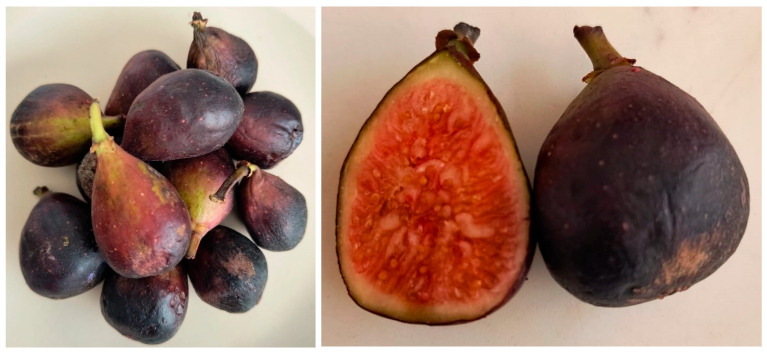
Ripe fruits “Black figs” belonging to the species *F. carica*.

The genus *Ficus* L. (belonging to the *Moraceae* family) comprises more than 850 species of angiosperms (commonly known as fig trees) distributed in different tropical and subtropical regions of the world. Most species grow wild in the eastern Mediterranean, Asia, Africa, Australia, and much of the Americas. Their fruits (known as figs) are infructescences with a thin, smooth skin, followed by a fleshy, edible substance (Figure 2) [7,8,9]. Among the most important species are *F. deltoidea*, *F. exasperata*, *F. sycomorus*, *F. religiosa*, *F. microcarpa*, *F. hirta* Vahl., *F. benghalensis*, *F. racemosa*, *F. elastica*, and *F. carica*, the latter four being the most significant worldwide [7,8,9]. Specifically, *F. carica* (Figure 2) is considered a very popular fruit crop whose figs (black, green, purple, and blue) are consumed by different populations around the world as fresh, dried, or dehydrated food, jams, sweets, or vacuum packaged [7,8,9]. A key characteristic of *F. carica* is its easy adaptation and spreading in different soil types, which has favored its domestication process, increasing the harvesting and constant consumption of its figs [7,8,9].

Nutritionally, *Ficus* L. genus contains large amounts of soluble dietary fiber, water (80%), amino acids (especially glutamine and aspartic acid), minerals (Cu, Mg, K, Mn, Fe, Zn, Ca, and Na), vitamins (A, C, B1, B2, and B6), starches and sugars (the most common being glucose, fructose, sucrose, and pectins), and organic acids (oxalic, quinic, malic, citric, and succinic) [7,8,9]. Among its main identified bioactive molecules are flavonoids (flavanols, flavones, flavonols, isoflavones, chalcones, anthocyanins), phenolic acids (hydroxycinnamic acids, hydroxybenzoic acids), terpenes (triterpenes, tetraterpenes, diterpenes, sesquiterpenes, monoterpenes), phytosterols, coumarins, hydroxybenzoates, phenylpropanoids, chlorins, pheophytins, mega-stigmans, chitinases, organic acids, fatty acids, amino acids, alkaloids, and glycosides [7]. Most studies agree and suggest that there are differences between wild and domesticated species in the phytochemical composition of plant parts (roots, flowers, stems, bark, leaves, seeds, latex, pulp, and fruits). These differences are attributed to environmental conditions (climate, humidity), soil type at cultivation sites, harvest time, and age of maturity of the shrubs, all of which undoubtedly modify their functional and therapeutic properties [7,8,9].

Given that the genus *Ficus* has more than 30 traditional ethnomedical uses, attributed to the combination of its bioactive compounds [7], a scientific search was carried out in the main electronic databases (PubMEd, SciELO, Latindex, Redalyc, BiologyBrowser, ScienceResearch, ScienceDirect, World Wide Science, Web of Science, academic journals, Ethnobotany, Scopus and Google Scholar), using and combining keywords such as genus *Ficus* L., plants or species of *Ficus*, antimutagenic, anticarcinogenic and antigenotoxic effect and/or capacity in order to collect information from published research (in vitro and in vivo) on its genoprotective potential. Unlike most scientific articles, which primarily describe the individual characteristics and properties of each species, this document compiles the largest collection of studies on *Ficus* plants described by various authors. Therefore, the aim of this manuscript is to encourage more detailed scientific research and expand studies on the protective capacity of these angiosperm plants to the genetic material.

## 3. Genoprotective Evidence

### 3.1. Antimutagenic Capacity

Given that genoprotective agents can act against one or more mutagens and/or carcinogens and through a combination of several mechanisms (that is to say, they have different antigenotoxic spectra of action), it is relevant to evaluate their usefulness, efficacy, and spectrum of action using different assays. Currently, several in vitro and in vivo assays exist to determine the genoprotective capacity of compounds; among the most prominent tests of recent decades are the bacterial mutation assay (Ames test), sister chromatid exchange, the evaluation of chromosomal aberrations (ChAb), the micronucleus assay (MN), and the alkaline comet assay (single-cell gel electrophoresis). In general, each test has its advantages and disadvantages, but the main quality sought is that it is a sensitive, rapid, and simple method capable of evaluating the genotoxic and antigenotoxic effect in somatic and germ cells [4,5,10,11]. In the case of the *Ficus* L. genus, some species have demonstrated significant antimutagenic potential due to their high content of bioactive compounds (especially flavonoids, coumarins, and tannins), which protect against DNA damage. Several studies (Table 1) suggest and indicate that extracts (obtained mainly from stem bark and leaves) prevent damage to genetic material and reduce the mutagenic effects of some genotoxic substances. The results and/or evidence published by various authors [7,8,9,10] support the traditional use of the *Ficus* L. genus for various ailments and suggest significant potential for the development of new medicinal products. Despite their diverse traditional ethnomedicinal uses, the fact that many varieties of their plants (domesticated or wild) have been consumed since ancient times, and that they remain an essential food source for the human population (especially the fruits of *F. carica*), studies on the antimutagenic potential of the genus are still in their early stages. Agabeili and Kasimova (2005) were the first researchers to analyze, both individually and in combination, the antimutagenic activity of extracts from *F. carica*, *Armoracia rusticana*, and *Zea mays* against various environmental xenobiotics. Their results showed a decrease in the level of N-methyl-N′-nitro-N-nitrozoguanidine-induced mutations in *Vicia faba* cells, in chlorophyll mutations in *Arabidopsis thaliana*, and in NaF-induced genetic alterations in rat bone marrow cells [12].

Probably the most frequently observed antigenotoxic mechanism is the antioxidant capacity (reduction in oxidative stress, “OXS”). However, the results of research conducted to date suggest other possible mechanisms (such as anti-inflammatory and mitogenic properties, reduction in DNA strand breaks, and decreased formation of MN and ChAb) [7,13]. Various fractions and/or extracts (mainly aqueous, methanolic and ethanolic) have demonstrated demutagenic effects (partial or total inactivation of mutagens through enzymatic or chemical interaction before the agent attacks the genes). Other scientific evidence, especially that related to anticarcinogenic activity (this will be discussed later), also suggests actions on the replication and repair processes of DNA damaged by mutagens and/or carcinogens, as well as activation of apoptosis, which could reduce alterations to the genetic material (bioantimutagenic effects) [10].

**Table 1:** Scientific evidence demonstrating the antimutagenic capacity of the genus *Ficus* L.

Extract/Plant Part	Type of Assay	Phytochemical Related to the Beneficial Effect	Objective and/or Methodology	Results and/or Conclusion	Ref.
***F. benghalensis***					
Methanolic/Stem bark	In vivo		The antimutagenic protection of the extract against cyclophosphamide (CP)-induced genotoxicity was evaluated in the bone marrow of Swiss-albino rats	Oral administration of the extract (doses of 250, 500, and 800 mg/kg) significantly decreased MN formation and chromosomal abnormalities in a dose-dependent manner	[14]
Aqueous/Stem bark	In vitro		The antimutagenic and antioxidant activities of the heat-treated extract against sodium azide (NaN3) in TA100 strains of *Salmonella typhimurium* and its potential to inhibit LPO were investigated	The Ames test confirmed an IC50 of 70.24 mg/mL. Significant antioxidant activity and high LPO inhibition (IC50 of 80.24 mg/mL) were also observed	[15]
Aqueous-and methanolic/Stem bark	In vitro		The objective was to determine the genoprotective effect of the extract against H_2_O_2_-induced DNA damage and repair in human neuroblastoma cells (SK-N-SH)	Pre-incubation with the extract attenuated cellular toxicity and increased cell viability affected by exposing them to H_2_O_2_ (MTT assay). It also significantly decreased the intensity of oxidative damage in SK-N-SH cells (Comet assay). It is concluded that the extract has relevant neuroprotective activity	[16]
***F. carica***					
Leaves	In vitro	Luteolin	The protective potential of this flavone against genotoxicity induced by an halogenated boroxine (therapeutic agent for treating skin disorders) was analyzed in human peripheral blood lymphocytes	The phytoconstituent (50 μM concentration) reduced the frequency of chromosomal aberrations analyzed by the cytokinesis-block micronucleus cytome assay. It also decreased cell proliferation and inhibited the cell cycle at the G1/S and G2/M points	[17]
Aqueous/Leaves	In vivo	Flavonoids	Using the comet assay and the micronucleus test in mice, the genotoxic effects of the plant species were determined under the influence of the specific climatic conditions (in both healthy and polluted areas) in the Asir region of Saudi Arabia	Extracts from plants grown in polluted soil produced greater cytotoxicity and a significant increase in the number of micronucleated cells. In contrast, extracts from plants grown in unpolluted environments showed normal values comparable to a control group. In conclusion, pollution has significant effects on these phytoconstituents and the genotoxic potential of *Ficus* species	[18]
Aqueous/Leaves	In vitro	Phenolic compounds	The authors hypothesized that the extract inhibited diethylstilbestrol (DES)-induced DNA damage. MCF10A human breast epithelial cells were treated with DES and three different concentrations of the extract (1, 10, and 100 μM). Subsequently, single-strand DNA breaks in the cells were analyzed using the comet assay	Their results indicated that exposure to the extract did not produce DNA damage. On the contrary, DNA strand breaks were attenuated. Possibly, a mechanism of action for cancer chemoprevention related to the active ingredients of the extract might exist	[19]
Methanolic/Leaves	In vivo	Catechin, luteolin-8-C-β-D-glucopyranoside, quercetin, quercetin-3-O-β-D-glucopyranoside, chlorogenic acid, and kaempferol-3-O-β-D-glucopyranoside	The protective potential of the extract against the hepatotesticular side effects and cisplatin (CP)-induced genotoxicity was evaluated	At the end of the experiment, it was clear that the extract attenuated the destructive effects of CP on the testicles, liver, and bone marrow due to the presence of a high amount of flavonoids. The chromatographic analysis identified six bioactive compounds. CP induced chromosomal abnormalities and MN; this phenomenon was reversed with the three doses of the extract (200, 400, and 600 mg/kg). Conclusively, this plant species is a promising candidate for mitigating the destructive effects of antineoplastic agents	[20]
*** F. palmata ***					
Aqueous/Stem bark (AESBFp)) and methanolic/Leaves (MELFp)	In vitro	Phenolic compounds	Since hypercholesterolemia-induced OXS is linked to the pathogenesis of atherosclerosis, the antioxidant and genoprotective effects of the extracts were evaluated. The study confirmed that both fractions have a high concentration of these phytoconstituents and a significant antioxidant capacity	The study confirmed that both fractions have a high concentration of these phytoconstituents and a significant antioxidant capacity. However, the Assay for Oxidative DNA Strand Breaks determined that AESBFp provided better protection against oxidatively damaged DNA. In conclusion, both extracts may play a relevant role in combating some diseases related to OXS, including aterosclerosis	[21]
*** F. adhatodifolia * and * F. obtusiuscula ***					
Aqueous/Leaves	In vitro	Flavonoids, coumarins, and tannins	Initially, a phytochemical analysis, a quantification of the phenolic profile, and a determination of the total phenolic content of the extracts were performed. Subsequently, the antioxidant activity (DPPH assay) and mutagenic potential were evaluated by means of the Ames test, both in the presence and absence of metabolic activation	Chromatographic techniques (TLC and UPLC-DAD-ESI/MS/MS) revealed the presence of various bioactive compounds; however, *F. obtusiuscula* exhibited higher concentrations. Worryingly, both species showed mutagenic activity of strain TA97 without metabolic activation and of strain TA102 with metabolic activation. This evidence suggests that the use of infusions made from these two plant extracts requires further investigation due to their potential pro-oxidant mutagenic effects	[22]
***F. religiosa***					
Aqueous and ethanolic/Leaves and Stem bark	In vitro		The genoprotective effect of the extracts on H_2_O_2_-induced oxidative DNA damage in human lymphocytes was analyzed using the comet assay	Results showed an increase in comet tail length when samples were treated with H_2_O_2_; this increase was reduced with all extracts. The highest antigenotoxic activity was observed with the ethanolic leaf extract	[23]
***F. deltoidea***					
Aqueous/Leaves	In vitro		Due to the lack of knowledge regarding the antimutagenic and cytoprotective potential of the plant species, these properties were evaluated using the extract on the TA 98 and TA 100 strains of the Ames test and the menadione-induced OXS in a V79 mouse lung fibroblast cell line	The results were that concentrations of the extract (up to 50 mg/mL) did not show mutagenic effects on the bacterial strains. Conversely, a decrease in the count of revertant colonies induced by 2-aminoanthracene was observed in both strains in the presence of metabolic activation. Pretreatment with the extract (50 and 100 µg/mL) also showed remarkable protection against menadione-induced oxidative oxidation in V79 cells. The FRAP assay confirmed its antioxidant capacity by reducing superoxide anion levels. It is suggested that this plant species may exert a chemopreventive effect against mutagenic and OXS-inducing agents	[24]
***F. erecta***					
Ethanolic/Leaves (EELFe)	In vitro In vivo		Given that scientific evidence on the safety of extracts from this plant species is limited, the genotoxicity of EELFe was determined using different tests	Using the bacterial reverse mutation assay (Ames), it was confirmed that in 5 bacterial strains (*Salmonella typhimurium* TA98, TA100, TA1535, TA1537 and *Escherichia coli* WP2uvrA), with a concentration of up to 312.5 μg/plate, the number of revertant colonies was not affected in the absence or presence of the metabolic activator S9. Likewise, the number of chromosomal aberrations did not increase up to a concentration of 31.3 μg/mL. Regarding the in vivo micronucleus test, the results showed that doses up to 2000 mg/kg did not influence the frequency of MN in the bone marrow of ICR mice	[25]

**LPO**: Microsomal lipid peroxidation, **OXS**: Oxidative stress, **DPPH**: 2,2-diphenyl-1-picrylhydrazyl radical scavenging capacity, **TLC**: Thin layer chromatography, **UPLC-DAD-ESI/MS/MS**: Ultra-high performance liquid chromatography plus a diode array detector and a mass spectrometer, **FRAP**: Ferric-reducing antioxidant capacity, **MTT**: 3-(4,5-Dimethylthiazol-2-Yl)-2,5-Diphenyltetrazolium Bromide assay.

**Figure 3:**
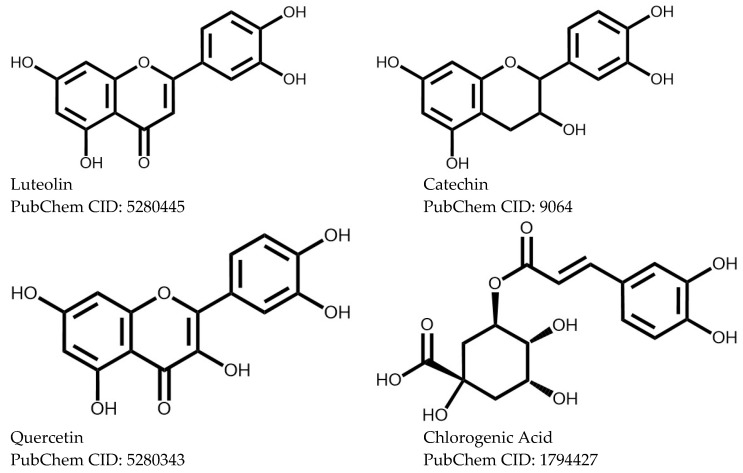
Isolated bioactive compounds with antimutagenic capacity.

As mentioned above, flavonoids, coumarins, and tannins are the main phytochemicals associated with the antimutagenic potential of the genus *Ficus*. Specifically, luteolin, catechin, luteolin-8-C-β-D-glucopyranoside, quercetin, quercetin-3-O-β-D-glucopyranoside, chlorogenic acid, and kaempferol-3-O-β-D-glucopyranoside have been isolated. Figure 3 shows some of their chemical structures.

### 3.2. Anticarcinogenic Capacity

Unlike the potential for antimutagenicity, the literature and electronic databases reviewed show a greater number of studies on the anticarcinogenic capacity of the genus *Ficus*. The first scientific evidence of these plants against cancer was obtained by Ullman et al. (1945, 1952) using latex extracts from *F. carica*. Injections of the extracts into albino rats demonstrated the inhibition of benzo[a]pyrene-induced sarcoma growth and a reduction in small tumors [26,27,28]. These results inspired the isolation and structural elucidation of a mixture of 6-O-acyl-beta-D-glucosyl-beta-sitosterol isoforms from the same type of latex, a bioactive compound that subsequently demonstrated antiproliferative activity in different tumor cell lines [29]. From these investigations, explorations into its anticarcinogenic capacity (ability of a substance to fight cancer by preventing the development, inhibiting the growth, or inducing the death of cancer cells) began in a significant way. To date, the most studied species are *F. exasperata*, *F. microcarpa*, *F. formosana*, *F. septica Burm*, *F. racemosa*, *F. salicifolia*, *F. elastica*, *F. religiosa*, *F. hispida*, *F. benjamina*, *F. virens*, *F. benghalensis*, and *F. carica* in relation to cancer and inflammation. In this regard, Li et al. (2004) isolated racemic acid from the bark of *F. racemosa* to determine its inhibitory effect against cyclooxygenase 1 and 5-lipoxygenase activity [30]. Similarly, aqueous extracts of its leaves have demonstrated anti-inflammatory activity in rat limb edema models induced by carrageenan, serotonin, histamine, and dextran [31]. Studies evaluated in bovine udder papillomatosis (benign tumors that can undergo malignant transformation to squamous cell carcinomas) corroborated that fig latex has an anti-inflammatory effect similar to salicylic acid in controlling this bovine pathology [32].

Some flavonoids from the stems of *F. formosana* Maxim. (apigenin, carpachromene, and norartocarpetin) have been observed to be cytotoxic (action of causing cell death by affecting their metabolism and/or replication) in several cancer cell lines (Human hepatoma, lymphoma, and acute monocytic leukemia) [33] and lectins from fig seeds cause leukocyte agglutination in patients with leukemia, but not in healthy individuals [34]. Among the most promising cytotoxic compounds from *Ficus* species are triterpenoids with carboxylic acid functional groups and phenanthroindolizidine alkaloids isolated from the aerial roots of *F. microcarpa* with evidence of this potential in human cancer cell lines (nasopharyngeal carcinoma HONE-1, oral squamous cell carcinoma KB, and colorectal carcinoma HT29) [35]. Also, alkaloids obtained from the leaves of *F. septica Burm* (ficuseptin-A, (+)-tylophorin, and a mixture of (+)-anthophin and (+)-isotylocrebrine) and from the stems of *F. hispida* (O-methyltylophorinidin) exhibit potent cytotoxic activity with average inhibitory concentration at 50% (IC_50_) values around 2 microM [36,37]. Table 2 summarizes the main results of the research demonstrating the promising anticancer activity of these angiosperms.

Recently, Laum et al., (2025) conducted a systematic review of laboratory studies on *Ficus* species traditionally used against cancer in the Philippines. They found 76 documents that included 5 species (*F. septica*, *F. elastica*, *F. congesta*, *F. concinna*, and *F. botryocarpa*) used locally, mainly in the form of leaf decoctions. In their results they identified bioactive compounds such as terpenoids, flavonoids and alkaloids, which show cytotoxic effects against various types of cancer through mechanisms such as cell cycle arrest, apoptosis, antioxidant modulation and inhibition of metastasis [57]. These species of Philippine origin, together with those previously mentioned (*F. exasperata*, *F. microcarpa*, *F. formosana*, *F. septica* Burm., *F. racemosa*, *F. salicifolia*, *F. religion*, *F. hispida*, *F. benjamina*, *F. virens*, *F. benghalensis* and *F. carica*), suggest and demonstrate the anticarcinogenic potential of the genus.

**Figure 4:**
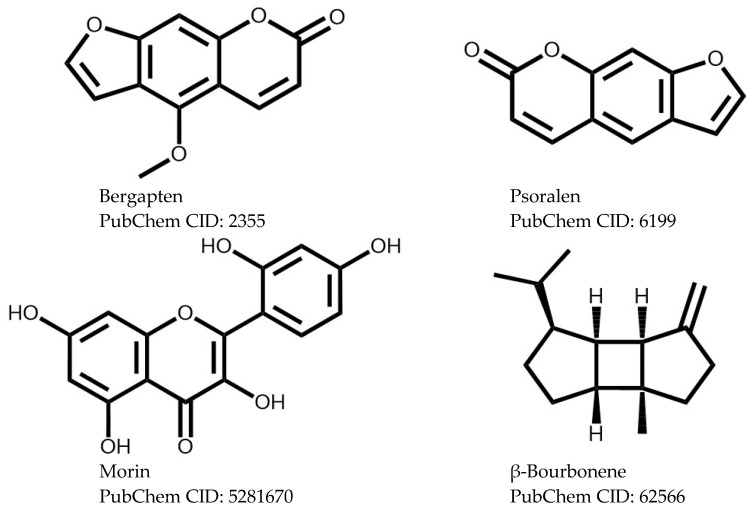
Isolated bioactive compounds with anticarcinogenic capacity.

Although archaeological evidence suggests that *Ficus* species have been domesticated and harvested for more than 11,000 years (before cereals) [7] and that their fruits are currently a common food, it is curious and surprising that most of the results on their biological activities are derived mainly from in vitro tests and animal evaluations. That is, few clinical studies have been developed, and they have primarily focused on the analysis of its activities, such as hypoglycemic, hypolipidemic, antiviral, anti-wart, and in skin healing [7,8,58,59,60]. Therefore, if the results of the research that exists to date suggest the anticarcinogenic capacity of these angiosperm plants, it would be relevant to begin controlled clinical studies; moreover, different authors consider the genus relatively safe (even at doses higher than 6000 mg/kg, in oral and intraperitoneal administrations), since apparently, the side effects that have been observed are related to skin irritation and/or allergies (pigmentation on arms and face), respiratory diseases (rhinitis, asthma), abdominal pain, and catharsis (evacuation of liquid stools) [7,8,61,62]. Figure 4 shows some of the isolated bioactive compounds with anticarcinogenic capacity.

## 4. Perspectives and Conclusions

This study synthesizes the most precise scientific evidence on the genoprotective actions of the genus *Ficus* L. Several authors agree that the most studied species are *F. deltoidea*, *F. benghalensis*, *F. racemosa*, *F. elastica*, and *F. carica*, out of 850 species belonging to the *Moraceae* family. In particular, the data presented in this document confirm that *F. carica* is the fig tree with the most research on its antimutagenic and anticarcinogenic potential (approximately 19 studies have been conducted in both areas of exploration).

Furthermore, a review of the presented results shows that most were obtained from uncharacterized crude extracts. Hence, it would be relevant to increase research on phytochemical standardization and bioactivity-guided identification of metabolites to specifically identify the bioactive compound responsible for these beneficial effects. Moreover, there are differences in the quantity and type of bioactive compounds present in plant parts (roots, flowers, stems, bark, leaves, seeds, latex, pulp, and fruits) between wild and domesticated species. In this regard, it can be mentioned that some authors consider that leaves contain more than 120 phytoconstituents, of which polyphenolic compounds predominate, while other researchers suggest that phenolic acids and flavonoids are more relevant in fresh and dried figs [7].

In some of the investigations shown, the responsible phytoconstituent was identified; such was the case of luteolin and morin, flavonoids present in the leaves and figs of *F. carica*, respectively [17,43], racemic acid obtained from the bark of *F. racemosa* [30], flavonoids from the stems of *F. formosana* (such as apigenin, carpachromene and norartocarpetin) [33], some lectins from fig seeds [34], triterpenoids with carboxylic acid functional groups and phenanthroindolizidine alkaloids isolated from the aerial roots of *F. microcarpa* [35], and different alkaloids (ficuseptin-A, (+)-tylophorin, O-methyltylophorinidine) obtained from leaves of *F. septica* Burm. and stems of *F. hispida* [36,37].

Similarly, the scientific results presented in our document indicate that the compounds primarily come from extracts of stem bark, leaves, figs, and latex. Therefore, it would be advisable to increase the number of studies using the roots, flowers, seeds, and pulp obtained from other species, especially when remembering that there is a difference in the phytochemical composition between wild and domesticated species, a situation that can be attributed to environmental conditions (climate, humidity), soil type of the cultivation sites, harvest time and age of maturity of the shrubs, which undoubtedly modifies the functional and therapeutic properties of the genus.

After reviewing and analyzing the 31 studies included in this document, we confirmed that all are preclinical in nature and, in some cases, suggest possible mechanistic processes. In this regard, it is worth remembering that preclinical studies constitute the basis of modern medicine by fulfilling several essential purposes, with the following standing out: a) obtaining mechanistic information by allowing detailed exploration of disease pathways at the cellular and molecular level, b) determining potential pharmacological targets by attempting to discover the therapeutic effect of different biological products and/or candidate drugs in controlled environments, c) evaluating the safety of the compounds and/or biological products, that is, toxicological studies in animal models that evaluate organ-specific toxicity, dosage thresholds and pharmacokinetics, and d) identifying the proof of concept, where, by conducting studies in animals evidence of a possible potential and/or clinical value is obtained (i.e., the possibility of developing Translational Studies, “Trials in humans”) [63].

However, despite the importance of preclinical studies, unfortunately, they often do not predict outcomes in humans due to certain drawbacks, such as (1) physiological and genetic differences between humans and animals that limit the predictive value of animal models, and the fact that (2) in vitro and in vivo models often simplify complex human conditions. That is, many diseases (such as cancer and neurodegeneration) involve multifactorial processes that are not fully captured in laboratory systems. (3) There are also problems of reproducibility in humans, given that in vitro and animal assays are often inconsistent due to the small sample size and lack of standardization, which can reduce the reliability of the results, as well as (4) ethical restrictions, where some specific human conditions cannot be fully modeled due to the ethical limitations of animal research. (5) Finally, there are regulatory obstacles, that is, the translation of laboratory findings into clinical protocols where strict regulatory approval is required, which generally delays progress [63].

Although several authors have suggested that the genus *Ficus* is a relatively safe plant species, since it rarely presents mild side effects (skin irritation, allergies, rhinitis, asthma, abdominal pain and catharsis) and does not induce other adverse toxic effects, even at doses higher than 6000 mg/kg, both orally and intraperitoneally [7,8,61,62], it would be relevant to increase the evaluations of its toxicological effects to consider exploring the possible potential benefits in controlled clinical studies, in particular, with those species that are commonly harvested and whose fruit is frequently consumed by different populations around the world as fresh, dried, dehydrated, canned, jams, sweets, and vacum packaged [7,8,9].

Finally, conducting further studies with other species and using different antimutagenic and/or anticarcinogenic assays, with the primary aim of establishing other antigenotoxic mechanisms of action (in addition to antioxidant potential and reduction in oxidative stress), would favor the identification of new genoprotective agents that could support the control and reduction in alterations to genetic material. In conclusion, there is still much to be done in scientific research on the genoprotective action of these angiosperms belonging to the *Moraceae* family.

## Figures and Tables

**Table 2:** Scientific evidence demonstrating the anticarcinogenic capacity of the genus *Ficus* L.

Extract/Plant Part	Type of Assay	Phytochemical Related to the Beneficial Effect	Objective and/or Methodology	Results and/or Conclusion	Ref.
***F. carica***					
Fig tree	In vitro	Latex	The researchers evaluated the effect of five concentrations of latex (0.125, 0.25, 0.5, 1.0, 2.5, and 5.0 mg/mL) on stomach cancer cells and peripheral blood mononuclear cells	After 72 h of treatment, inhibition of cancer cell line proliferation was observed without any cytotoxic effect on normal human cells. The greatest inhibition occurred at the 5.0 mg/mL concentration, and it was suggested that the therapeutic benefit was attributable to the presence of its proteolytic enzymes	[38]
Dried fruit extract (Figs)	In vitro In vivo		Due to the commercial demand for nanoparticles in the medical field, silver particles were synthesized from a AgNO_3_ solution using a dried fruit extract as a reducing and stabilizing agent	The spherical nanoparticles, ranging in size from 54 to 89 nm, made visible a significant cytotoxic effect on MCF7 cell lines. Furthermore, acute toxicity studies in animals indicated they were safe for oral administration	[39]
Fig tree	In vitro	Latex	A review of compounds derived from natural products with antiglioblastoma activity reported that latex induces let-7d expression and acts on the epithelial–mesenchymal transition of the HMGA2 gene in glioblastoma multiforme (GBM) cell lines, causing cell death by increasing the therapeutic potency of temozolomide	In consequence, the use of natural compounds may be a novel strategy for treating GBM by altering angiogenesis, metastasis, and microRNA expression	[40]
Fig tree	In vivo	Latex	Considering previous studies on latex, its beneficial properties on breast cancer growth, hematological parameters, and histopathology were analyzed in rats treated with 7,12-dimethylbenz(a)anthracene	Latex pretreatment significantly decreased the size of tumors induced by the carcinogen, as well as hematocrit, hemoglobin, and red blood cell counts; while blood parameters such as platelets, leukocytes, and white blood cells were increased, latex is suggested as a favorable anticarcinogenic agent that does not affect hematological factors	[41]
Aqueous/Leaves	In vitro	Bergapten and psoralen	This study aimed to analyze the anticancer effects of the extract on the triple-negative breast cancer cell line MDA-MB-231	The results showed a significant antiproliferative effect and an increase in the expression of genes that promote apoptosis. Likewise, the treated cells showed a higher proportion in the S phase and an altered expression of cyclin-dependent kinases, demonstrating cell cycle arrest in that phase, in addition to reduced cell mobility, essential for metastasis. On the other hand, two of the active components of the extract showed the same anticancer effects, suggesting that both the extract and these bioactive compounds could be considered as chemopreventive agents against triple-negative breast cáncer	[42]
Figs	In vivo	Morin	The molecular basis of the way in which this flavonoid contributes to chemoprevention was explored, focusing on the NF-κB signaling pathway. The effect of this phytochemical on 1,2-dimethylhydrazine-induced carcinogenic events was analyzed in a rat model of colon cancer. Inflammation, apoptosis, mRNA expression (using quantitative PCR, or “qPCR”), and protein expression (by immunohistochemistry) were analyzed	Pretreatment with the flavonoid reduced the activity of the NF-κB via and decreased some inflammatory mediators (COX-2, IL-6, tumor necrosis factor alpha “TNF-α”, and prostaglandin E2). Additionally, an alteration in the BAX/BCL2 ratio was observed, favoring the apoptotic process in rats treated with the carcinogen. That the flavonoid can act as a potent anti-inflammatory and pro-apoptotic agent for the prevention of colon cancer could also be confirmed	[43]
Methanolic/Figs and leaves	In vitro	Polyphenols	Although *F. carica* is rich in these phytoconstituents, its antioxidant and anticancer activities have not yet been fully characterized. The genoprotective impact of the extracts on cell proliferation, apoptosis, and necrosis in Huh7it hepatocellular carcinoma cells was determined	Both extracts showed potent antioxidant activity (DPPH radical). The MTT assay indicated that the leaf and fruit extracts showed IC50 values greater than 653 μg/mL and 2000 μg/mL, respectively. The flow cytometry analysis revealed a higher percentage of apoptosis and necrosis of Huh7it cells with the leaf extract. The genoprotective benefit was attributed to these bioactive compounds	[44]
Aqueous and methanolic/Leaves	In vitro	Flavonoids	The inhibitory activity of enzymes related to diabetes mellitus and Alzheimer’s disease was investigated, as well as the anticancer and antioxidant effects of the extracts. DPPH and ABTS assays suggested that both extracts possess strong antioxidant activity	The methanolic extract was confirmed to have greater anticancer potential in MDA-MB-231 cells. Finally, both extracts showed strong inhibitory activity against α-glucosidase and α-amylase; however, the aqueous extract evidenced the greatest effect. It is concluded that the therapeutic benefits of the extracts are related to the presence of their phytoconstituents	[45]
Chloroform, Hexane, ethyl acetate and methanol/Látex	In vitro In vivo	Lupeol acetate and lupeol palmitate	Iranian researchers prepared four latex fractions using different solvents. Afterwards, they studied the cytotoxic effect of each fraction and selected the most effective one for apoptosis assays, in vivo acute toxicity studies, and phytochemical analysis using column chromatography	The chloroform fraction was the most effective for the HepG2 and NIH cell lines. Flow cytometry revealed cells in the apoptotic phase, and phytochemical analyses confirmed the presence of two phytoconstituents. Since a dose of 2 g/kg of the extract did not cause death in the animals, this fraction could be considered a candidate for use as a chemopreventive agent	[46]
Figs, Leaves and Stem bark	Molecular docking and dynamic simulation	β-bourbonene	Although bioactive compounds have been isolated from *F. carica* and some of their pharmacological properties have been analyzed, their anticancer mechanisms remain unknown. Therefore, the objective of this study was to understand the possible mechanisms of action through molecular docking and dynamic simulation	The binding affinity of 68 bioactive compounds to target receptors was explored, such as cyclin-dependent kinase 2, cyclin-dependent kinase 6, topoisomerase-I, topoisomerase-II, B-cell lymphoma 2, and vascular endothelial growth factor receptor 2. In silico toxicity studies revealed that 13 compounds possess acceptable pharmacological properties. A tricyclic sesquiterpenoid hydrocarbon showed the best binding affinity to most of the pharmacological targets	[47]
Ethanolic/Leaves	In vitro		The researchers explored the antioxidant and anticancer properties of the extract	The results showed a potent inhibitory effect against HepG2 and human laryngeal carcinoma (Hep-2) cell lines (80% and 67%, respectively). The DPPH assay indicated significant antioxidant activity (76%) at a concentration of 1 mg/mL. These results support the health benefits of this traditionally used medicinal plant	[48]
Fig tree	In vitro	Latex	For the first time, latex was encapsulated using cellulose acetate (CA) and poly(ethylene oxide) (PEO) polymers via electrospinning method (Fig@CA/PEO	The nanofibers were effective against colon (Caco) and liver (HepG2) cancer cells, with IC50 values of 24 μg/mL. They also evinced mechanistic effects on apoptotic oncogenes, characterized by the overexpression of BCL2 and p21, along with the underexpression of p53 and TNF-α. In addition, they displayed significant antioxidant activity in DPPH uptake. These results demonstrate that Fig@CA/PEO nanofibers represent a promising alternative to traditional chemotherapy	[49]
Aqueous/Figs	In vitro		Building on the previous study, cerium oxide nanoparticles were synthesized using a green solution combustion method with the extract	The nanoparticles exhibited dose-dependent redox activity, resulting in a reduction in cell viability (approximately 49%) at a concentration of 50 μM. Fluorescence imaging showed a dose-dependent duality (“antioxidant at low concentration, pro-oxidant at high concentration”), consistent with mitochondrial damage and ATP depletion. Moreover, it induced remarkable degradation (95%) of methylene blue under visible light, indicating its significant potential for therapeutic applications	[50]
Phenolic/Latex	In vitro	Syringic acid	For the first time, changes in total phenolic content (TPC), phenolic profile, antioxidant activity, and anticancer potential against cervical cancer and colorectal cancer cell lines of the extract were analyzed during in vitro gastrointestinal digestions	The digestion process significantly decreased TPC and antioxidant activity (CUPRAC method). Neither the digested nor the undigested fractions showed cytotoxic activity against normal cells. However, the anticancer activity evaluated in the cell lines decreased with digestion. At the end of the study, a phenolic compound was identified	[51]
***F. carica* and *F. salicifolia***					
Fig tree	In vitro	Latex	When comparing the genoprotective activity of the latex of 2 *Ficus* species, the findings were that both fig trees a) possess antiproliferative effects (MTT assay), b) through a wound-healing assay, the antimetastatic effect of the species was demonstrated by maintaining the size of the wound compared to untreated cells, and c) using MDA-MB-231 cells derived from triple-negative breast cancer, the cytotoxic effect of the plants was confirmed	The mechanism of action appears to be related to the decreased expression of ERK2, CREB, and AKT2 after treating MDA-MB-231 cells; however, RT-PCR revealed a decrease in the expression of these vias in cells treated with *F. carica*, while in cells treated with the other species, the selected genes showed an increase in their transcriptional expression. In conclusion, both species have anticancer potential but differ in their toxicity level and molecular mechanisms of action	[52]
***F. benghalensis***					
Ethanolic, methanolic, ethyl acetate, and acetone	In vitro	Latex	The study analyzed the antiproliferative activity of the extracts in different cancer cell lines (Human breast MDA MB 231, colorectal HCT116, and neuroblastoma IMR 32) using the MTT assay	After analyzing all the extracts, the ethanolic extract proved to be the most effective against the IMR 32 and HCT116 cell lines, while the ethyl acetate extract was effective against MDA-MB 231 cells	[53]
***F. benghalensis**,** F. religiosa**,** F. elastica* **and ***F. **virens*****					
Methanolic and hexane/Leaves	In vitro	Carvacrol, phytol, tocopherol, benzophenone, dibutyl phthalate, and lycopersene	The aim of this research was to determine the phytochemical constituents and evaluate the antioxidant activity, protective potential against DNA damage, and anticancer properties of two extracts obtained from four common *Ficus* species. The methanolic fraction of *F. virens* contained the highest amount of flavonoids, while the hexane fraction of *F. religiosa* contained the highest amount of tannins. The lowest amount of phytochemicals was obtained from *F. elastica*	The DPPH and ABTS assays confirmed that the methanolic fraction of *F. benghalensis* exhibited the best antioxidant potential, while the same fraction from *F. virens* showed the greatest ferric-reducing power. The viability of normal mammary cells was not affected by the methanolic fraction of the plant species; however, cancer cell survival decreased with *F. benghalensis* at 5 μg/mL. Finally, GC-MS analysis of the methanolic fraction of all species revealed the presence of different phytoconstituents. This group of results supports the fact that the leaves of the analyzed *Ficus* species are a rich source of phytochemicals with nutraceutical potential	[54]
***F. hispida***					
Methanolic/Figs	In vitro	Isoflavones, coumarins, caffeoylquinic acids, phenols, and steroidal glycosides	Initially, 19 phytochemicals were isolated from the extract to evaluate both their inhibitory activity against Epstein–Barr virus early antigen (EBV-EA) activation induced by 12-O-tetradecanoylphorbol 13-acetate in Raji cells and their cytotoxic potential in 7 human cancer cell lines (HL60, A549, SKBR3, KB, HeLa, HT29, and HepG2) and a normal cell (LO2) using the MTT method. The apoptosis-inducing activity and DNA fragmentation activity of the compounds with the highest cytotoxicity were determined by flow cytometry	Five compounds (with an isowighone hydrate structure) showed potent inhibitory effects on EBV-EA induction. Five other phenolic compounds were cytotoxic against six cell lines. Only one phytochemical was active against caspases 3, 8, and 9, inducing apoptosis in HL60 cells. The conclusion was that the extract contains different phytoconstituents that may be valuable chemopreventive and anticancer agents	[55]
***F. benjamina***					
Chloroform/Leaves	In vitro		The study was conducted to examine the cytotoxicity of the plant species against human embryonic stem cells (HEK293T). Initially, the extract was separated into subfractions A, B, C, D, and E using thin-layer chromatography	Both the whole extract and the subfractions showed selective cytotoxicity toward HEK293T cells, with minimal impact on normal cells. The results also revealed that the extract had a more favorable therapeutic index than the anticancer drug bortezomib. Thus, *F. benjamina* is a promising candidate for cancer therapy research	[56]

**ABTS**: Azino-bis-tetrazolium sulfate radical scavenging capacity, **DPPH**: 2,2-diphenyl-1-picrylhydrazyl radical scavenging capacity, **FRAP**: Ferric-reducing antioxidant capacity, **TEAC**: Trolox equivalent antioxidant capacity, **MTT**: 3-(4,5-Dimethylthiazol-2-Yl)-2,5-Diphenyltetrazolium Bromide assay, **GC-MS**: Gas Chromatography coupled to Mass Spectrometry, **RT-PCR**: *Reverse Transcriptase Polymerase Chain Reaction*, **CUPRAC: Cupric Reducing Antioxidant Capacity.**

## Data Availability

Not applicable.
